# Contemporary Evolutionary Divergence for a Protected Species following Assisted Colonization

**DOI:** 10.1371/journal.pone.0022310

**Published:** 2011-08-31

**Authors:** Michael L. Collyer, Jeffrey S. Heilveil, Craig A. Stockwell

**Affiliations:** 1 Department of Biological Sciences, North Dakota State University, Fargo, North Dakota, United States of America; 2 Department of Biology, Western Kentucky University, Bowling Green, Kentucky, United States of America; 3 Biology Department, SUNY College at Oneonta, Oneonta, New York, United States of America; University of York, United Kingdom

## Abstract

**Background:**

Contemporary evolution following assisted colonization may increase the probability of persistence for refuge populations established as a bet-hedge for protected species. Such refuge populations are considered “genetic replicates” that might be used for future re-colonization in the event of a catastrophe in the native site. Although maladaptive evolutionary divergence of captive populations is well recognized, evolutionary divergence of wild refuge populations may also occur on contemporary time scales. Thus, refuge populations may lose their “value” as true genetic replicates of the native population. Here, we show contemporary evolutionary divergence in body shape in an approximately 30-year old refuge population of the protected White Sands pupfish (*Cyprinodon tularosa*) resulting in a body-shape mismatch with its native environment.

**Methodology/Principal Findings:**

Geometric morphometic data were collected from *C. tularosa* cultures raised in experimental mesocosms. Cultures were initiated with fish from the two native populations, plus hybrids, in high or low salinity treatments representing the salinities of the two native habitats. We found that body shape was heritable and that shape variation due to phenotypic plasticity was small compared to shape variation due to population source. *C. tularosa* from the high salinity population retained slender body shapes and fish from the low salinity population retained deep body shapes, irrespective of mesocosm salinity. These data suggest that the observed divergence of a recently established pupfish population was not explained by plasticity. An analysis of microsatellite variation indicated that no significant genetic drift occurred in the refuge population, further supporting the adaptive nature of changes in body shape. These lines of evidence suggest that body shape divergence of the refuge population reflects a case of contemporary evolution (over a 30-year period).

**Conclusions/Significance:**

These results suggest assisted colonization can introduce novel, and/or relaxed selection, and lead to unintended evolutionary divergence.

## Introduction

Contemporary evolution has important implications for conservation biology, as the same anthropogenic factors driving the current extinction crisis have been shown to be associated with a variety of cases of contemporary evolution (evolution over one to a few hundred generations) [1]–[4]. Additionally, traditional and emerging tools used by conservation biologists such as captive breeding, assisted colonization and creation of population refuges might alter evolutionary trajectories [5]–[6]. The pupfishes (*Cyprinodon* spp.) of southwestern North America are an ideal system for studying contemporary diversification because refuge populations are often established to provide sources for future re-colonization in the event of a catastrophe in a native site [7]–[12]. Thus, the evolutionary divergence of such refuge populations is likely to diminish their conservation value [5], [9]. Despite the potential impact, little research has investigated evolutionary divergence in refuge populations, although research examining the tempo and direction of evolutionary divergence in refuge populations can provide an understanding of the evolutionary responses of populations to local environments [13], [14].

Previous work has shown that body shape in the White Sands pupfish (*C. tularosa* Miller and Echelle) correlates with environmental salinity for both native and refuge populations [11], [12]. White Sands pupfish are classified as Threatened by the State of New Mexico, USA, and occur in four localities. Two native populations occur at Malpais Spring and Salt Creek, presumably isolated following the desiccation of Pleistocene Lake Otero approximately 3,000 to 5,000 years ago [15], [16]. These two populations occupy very different habitats, especially in terms of salinity and water flow. Malpais Spring is a brackish spring (typically 3.5‰) with no appreciable flow, whereas Salt Creek is a highly saline creek (25–80‰), which experiences greater variation in water flow [11], [17]. The native populations, which have been recognized as two Evolutionarily Significant Units of *C. tularosa*, are genetically differentiated at a level similar to divergence observed among recognized subspecies of pupfish [18].

Two other populations were introduced circa 1970 from transfers of Salt Creek fish [17], [18]. One population was established in Lost River, a saline habitat similar to Salt Creek. The other population was established in Mound Spring, a brackish habitat like that of Malpais Spring. These populations are putative refuge populations of the Salt Creek native strain.

In a previous study [11], a morphometric analysis revealed that pupfish in the saline habitats had more slender body shapes and pupfish in brackish springs were deep-bodied. These findings were consistent with the observation that euryhaline fishes occurring in saline habitats typically have more slender body shapes than fish occurring in comparatively less saline habitats [15], [19]. Salinity increases the density as well as the dynamic and kinematic viscosities of the water; smaller drag coefficients – achieved by streamlined body shapes – offset increased fluid density for fish moving through saline water [20]. Interestingly, fish introduced from Salt Creek to Mound Spring became the most deep-bodied population over a period of only 3 decades (30–60 generations) [11]. The previous work, however, was unable to identify whether differences in body shape were the result of phenotypic plasticity or contemporary evolutionary divergence.

**Figure 1:**
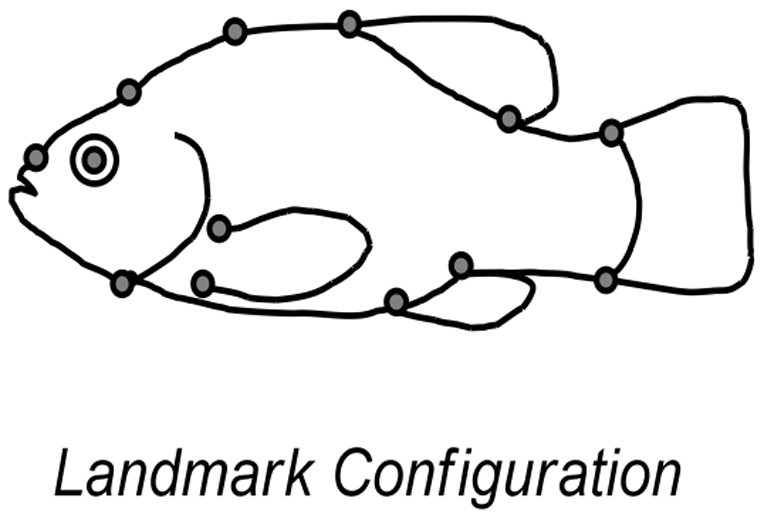
Landmark configuration used for shape analysis. Landmarks are described in [11].

Here, we analyze morphometric data (Fig. 1) collected from *C. tularosa* raised in experimental mesocosms from a common garden experiment. Geometric morphometric data [21], [22] were collected in the same manner as the previous study [11] from F1 generation pupfish cultured in the experimental mesocosms, which simulated the salinity differences between the two native habitats: high salinity (35‰, like Salt Creek and Lost River) and low salinity (3.5‰, like Malpais Spring and Mound Spring). Pupfish cultured in the mesocosms included native strains and hybrids, each subjected to both high and low salinity levels. Mesocosm cultures allowed us to evaluate the importance of population origin and environment on body shape, and thus, allowed us to evaluate if the shape divergence of the Mound Spring refuge population could be explained by phenotypic plasticity, or represented a case of contemporary evolutionary divergence. Additionally, we assessed if genetic drift could explain such divergence by evaluating variation at 8 selectively-neutral genetic markers from wild-caught fish.

## Materials and Methods

### Experimental populations

A “common garden” study was conducted at Holloman Air Force Base, New Mexico and included 36 mesocosms housing four experimental populations (produced from the four possible hybrid crosses between Salt Creek and Malpais Spring males and females) at two salinity levels: 3.5‰ (low) and 35‰ (high). These values fall within the salinity ranges found at the brackish springs (Malpais Spring and Mound Spring) and the saline creeks (Lost River and Salt Creek), respectively, where White Sands pupfish occur. Pools containing pure Salt Creek fish (henceforth denoted ‘SS’; each letter representing, in succession, the female and male origin for experimental fish) and Malpais Spring (henceforth ‘MM’) fish were replicated 12 times each, with 6 replicates assigned to each salinity treatment. Twelve replicates of hybrids were similarly cultured; however, 6 replicates were SM crosses and 6 were MS crosses (allowing consideration of maternal effects), with three replicates of each cross assigned to each salinity treatment.

Mesocosms were plastic pools approximately 1.5 m in diameter and 30 cm deep, provided with aquarium gravel and artificial grass for breeding substrate. Adult parental fish were collected from Salt Creek and Malpais Spring in the summer of 1996 and introduced to acclimation pools where salinity was gradually increased or decreased to the appropriate experimental conditions. Following acclimation, each experimental pool was stocked with 20 parental fish (10 male and 10 female) in July 1996. Fish were fed *ad libitum* twice a day with flake food and twice per week with brine shrimp nauplii. Parental fish were removed when F1 offspring approached reproductive size, at 13 months. The experiment was terminated at 17 months, and fish (>15 mm) were sacrificed in ice water, fixed in 10% formalin, and preserved in 70% ethanol. The mesocosm population sizes of first generation adult fish (>15 mm) are provided in Table S1. Two experimental pools did not produce offspring. Preserved specimens from this study were used to compare population and environmental sources of shape variation.

### Shape data

Preserved fish were photographed in 2002. Landmark coordinates were collected (by MLC) on the left lateral surface from 541 individual specimens from 34 successful mesocosms (Appendix S1; Table S1), without knowledge of population source or salinity. Data collection methods and photography followed the methods described in [11]. The Cartesian coordinates from digitized landmark configurations (Fig. 1) contain both shape and non-shape (i.e., size, orientation, and position) data. Non-shape variation was held constant with a generalized Procrustes analysis (GPA) [23], which centers and scales each configuration to unit size, and rotates configurations through a generalized least squares superimposition method to minimize the variation among landmarks. For some analyses, the “aligned” configurations were converted to shape variables by first finding the partial warps of the data set through a thin-plate spline analysis (TPS) [24], then performing a principal component analysis (PCA) on partial warp scores to produce relative warps. Relative warps are frequently used to describe shape variation among groups and the association of shape and other variables (e.g., size) with multivariate linear models, e.g., [11], [25], [26]; however, we followed the suggestion of [27] to subsequently align principal components based on variation among mesocosm types. This procedure rotates the morphospace to reveal the greatest among-group variation along the first principal component. Because mesocosm types are a product of both salinity and population sources, this “among-group” PCA allowed us to determine which factor was more prominent in explaining among-mesocosm type shape variation (through analyses explained below).

**Figure 2:**
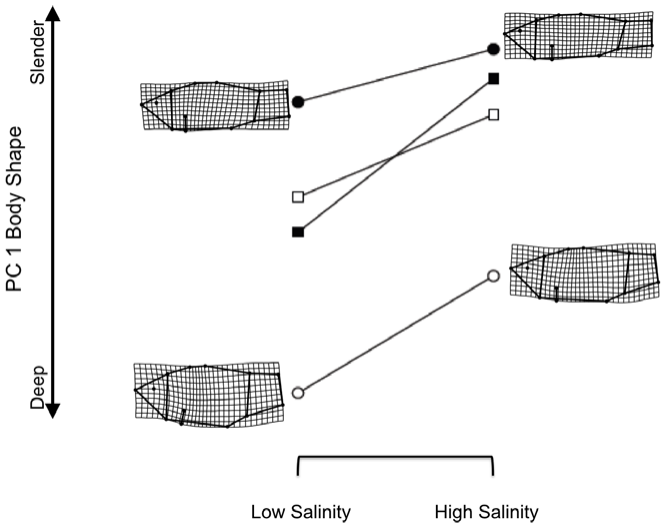
Graphical representation of shape variation for male *C. tularosa*. Values are shape means on the first principal component (PC) of among-group shape variation (representing 69.9% of among-group variation). Groups are the different source populations raised in either low or high salinity. Circles represent native crosses and squares represent hybrids. Solid symbols represent mesocosms that used Salt Creek females for the cross; open symbols represent mesocosms that used Malpais Spring females. Lines indicate reaction norms of shape change for the same population type introduce to high and low salinity environments. Deformation grids are scaled 3×, and are presented to facilitate an understanding of shape differences.

**Figure 3:**
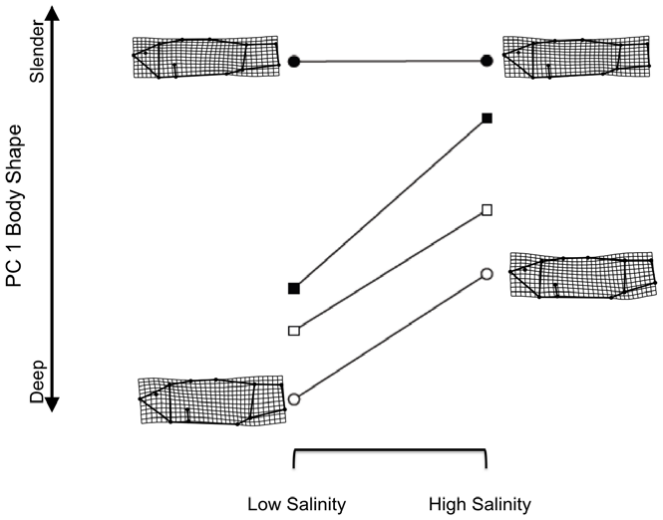
Graphical representation of shape variation for female *C. tularosa*. All information is the same as in Figure 1, except that the first PC represents 50.9% of the total variation.

For our analyses, all landmark configurations were adjusted to remove pool effects (Appendix S1 in the Supporting Information) and specimen size was always treated as a covariate of shape. We measured specimen size as the log of centroid size (CS; the square root of summed squared distances of landmarks from the configuration centroid) [24]. Shape means from mesocosm types (population source by salinity groups) were projected onto the first principal component of among-group shape variation for ease of interpretation and analysis, and deformation grids were generated for corresponding landmark configurations to visualize shape differences. Reaction norms for populations were plotted using means of mesocosm types to understand shape differences and how phenotypic plasticity compared to population differences in shape (Figs. 2 and 3). Shape means were also projected onto the first two principal components and reaction norms were visualized as two-dimensional vectors. These plots are presented in the Supporting Information (Figs. S1 and S2). GPA was performed for all fish using the software, tpsRelw (ver. 1.45) [28], but male and female configurations were analyzed separately because of known sexual dimorphism in shape [11]. Deformation grids were generated using tpsSplin (ver. 1.4) [29].

### Statistical Analyses

All morphometric data were analyzed with respect to the first principal component of among-treatment shape variation (i.e., univariate analysis of the major aspect of shape variation) and with the full set of shape variables (i.e., multivariate analysis of scores from 22 principal components), but results were largely consistent with either approach. Independent variables for models used to consider shape variation included the log of specimen centroid *size*, *salinity* (high or low), and *population source* (italicized words will henceforth identify model effects). There were four different levels of *population source*, based on the different hybrid cross types. This allowed us to evaluate maternal effects by evaluating if body shape varied according to hybrid direction (MS or SM). Alternatively, if maternal effects were not evident, *population source* could be described by three levels: pure Malpais Spring, pure Salt Creek, and hybrids (MS and SM).

We used two methods to consider the relative importance of components of shape variation for experimental populations, for both univariate and multivariate shape variables. First, Akiake's [30] information criterion (AIC) was used to compare different shape models. (We adjusted the computation of AIC values for multivariate data [31] to make them more comparable to interpretations normally made for univariate models; see Appendix S1 in the Supporting Information.) Based on the outcome of model comparisons, we next performed non-parametric permutation procedures for analyses of variance. This method is analogous to a traditional ANOVA, but is not sensitive to inferential errors that could be made with inappropriate degrees of freedom (Appendix S1) and worked equally well with univariate and multivariate shape data. Model comparisons and permutation procedures were performed using the statistical program R (ver. 2.12.1) [32].

### Comparison of results to previous morphological analyses

Because this study used the same landmark configuration as the previous study [11], the Procrustes distance [24] of the SS plasticity vector (between high and low salinity) can be used to predict the shape divergence of the Mound Spring population due to phenotypic plasticity of Salt Creek fish alone, provided shape differences between native populations were comparable between the two studies. We calculated the Procrustes distance between average shapes of SS fish raised in high salinity and MM fish raised in low salinity, in the mesocosms, and qualitatively compared this to the Procrustes distances between the average shapes of Salt Creek and Malpais Spring fish sampled from wild populations [11]. We then qualitatively compared the Procrustes distance between high and low salinity averages for SS fish raised in mesocosms to the Procrustes distance between average Salt Creek and Mound Spring fish sampled from wild populations. The former is a proxy for expected shape change as a result of phenotypic plasticity for Salt Creek fish introduced to a less saline environment.

### Genetic data

Forty fish were caught by minnow-trapping and seining during March, 2003, at Salt Creek, below Range Road 316, and Mound Spring, upper pool. These are the same locations sampled for assessment of body shape analysis of the wild populations (described in [11]). Fish were subsequently sacrificed (500 mg/L MS-222), frozen, and stored at −80°C upon return to the laboratory.

Whole genomic DNA was extracted from fin tissue using DNeasy kits (Qiagen, Valencia CA) and stored at 4°C. Eight microsatellite loci previously shown to be polymorphic in the Salt Creek population of *C. tularosa* were used to assess genetic differentiation: WSP2; WSP23, WSP24, WSP25, WSP33, WSP34; AC23, and GATA02 [16], [33], [34] (Table 1).

**Table 1:** Primer information and running conditions for loci used to examine population structure in *Cyprinodon Tularosa*.

Locus	Motif	[fwd primer](uM)	Annealing T	cycles
AC23	(CA)_n_	0.2	50;53	5;30
GATA2	(GATA)_n_	0.4	50;53	5;30
WSP2	Compound	0.2	55	30
WSP23	(TG)_n_-G-(GT)_n_	0.04	52	40
WSP24	(CA)_n_	0.2	55	32
WSP25	Compound	0.02	55	32
WSP33	(GT)_n_	0.04	55	32
WSP34	(TG)_n_	0.04	61	32

Complete motifs for loci with compound microsatellites available from the authors upon request.

Amplification reactions were performed in 25 ul volumes using 2.5 ul 10× PCR buffer, 200 uM dNTPs, 0.875 units AmpliTaq Gold polymerase (Applied Biosystems), 1 ul template DNA, 0.25 uM unlabeled reverse primer, and dye-labeled forward primer (concentrations listed in Table 1). The annealing temperatures and number of cycles varied for each primer set (Table 1). Automated fragment analysis was performed on a Beckman Coulter CEQ8000, using 600 size-standard (0.5 ul). Tests for Hardy-Weinberg Equilibrium (HWE) and linkage disequilibrium, paired F-statistics and exact tests of sample differentiation were performed for each subpopulation using Arlequin (ver. 3.0) [35].

## Results

### Morphological analyses

Model comparisons indicated for both males and females, and for both univariate and multivariate shape data, that *population source*, *salinity*, and their interaction were important sources of shape variation, but maternal effects had limited importance (Table 2). The *population source*×*salinity* interaction was also only marginally important in males, as a model lacking the interaction was nearly as good as a model containing it, based on differences in AIC scores less than 2. (If Δ AIC≤2, competing models should be considered equally viable, as recommended by [36].).

**Table 2:** Comparison of different models of pool-adjusted shape variation.

		Males		Females	
Model	*k*	AIC	AIC*	AIC	AIC*
Size only	2	190.7	19.4	195.6	23.9
S	3	173.7	14.0	180.0	18.6
P	4	56.9	7.9	58.8	10.0
P+ME	5	55.1	6.6	41.0	8.6
S+P	5	3.5	**1.9**	21.4	3.6
S+P+ME	6	5.1	**0.8**	12.9	2.6
S+P+PxS	7	**0**	**1.0**	**1.8**	**1.2**
S+P+(P+ME)×S	9	**1.1**	**0**	**0**	**0**

Model terms include salinity (S), population without respect to hybrid distinction (P) or with respect to hybrid distinction (i.e, maternal effects, P+ME), plus interactions. All models use specimen size as a covariate. AIC* indicates that the AIC is modified for multivariate shape data (see Supporting Information). Bolded values indicate that models are potentially equally viable [36].

Univariate and multivariate analyses of variance (ANOVA and MANOVA, respectively) largely confirmed the results of the model comparisons (Table 3). For both males and females, *population source* (containing maternal effects) was the most prominent source of shape variation, especially compared to *salinity*. For males, the *population source*×*salinity* interaction was not significant, indicating that shape differences between high and low salinity were rather consistent among the four crosses (Fig. 2). For females, however, the interaction was significant, and resulted from SS females retaining a slender body shape, even in low salinity (Fig. 3). For both males and females, MM fish retained deep body shapes and SS fish retained slender body shapes, compared to each other, and shape differences between low and high salinity environments, if any, were small compared to population differences. Hybrids were intermediate in shape, in both environments, indicating body shape was heritable.

**Table 3:** ANOVA and MANOVA statistics for univariate shape (PC 1) and multivariate shape (PCs 1–22) data, respectively, for both males and females.

	Males						Females					
	ANOVA			MANOVA		ANOVA			MANOVA		
Source	*Sums of Squares*	*R* ^2^	*P*	*Sums of Squares*	*R* ^2^	*P*	*Sums of Squares*	*R* ^2^	*P*	*Sums of Squares*	*R* ^2^	*P*
Population (cross-type)	0.0381	0.485	0.0001	0.0488	0.123	0.0001	0.0244	0.390	0.0001	0.0325	0.073	0.0001
Salinity	0.0074	0.094	0.0001	0.0112	0.028	0.0001	0.0035	0.056	0.0001	0.0122	0.027	0.0001
log(*CS*)	0.0048	0.061	0.0004	0.0121	0.030	0.0001	0.0004	0.006	0.1697	0.0141	0.032	0.0001
Pop×Sal	0.0013	0.016	0.3199	0.0056	0.014	0.1665	0.0019	0.031	0.0206	0.0075	0.017	0.0052
Residuals	0.0270	0.344		0.2440	0.613		0.0323	0.517		0.3802	0.851	

*Sums of squares* are calculated as the trace of the sum of squares and cross-products matrix for the associated effect. *P*-values were determined from empirical distributions of random *Sums of Squares* statistics (see Appendix S1 in the Supporting Information for more details).

The significant results using the univariate shape scores are not surprising because the alignment of principal components of shape was influenced by the *population source*×*salinity* treatment differences. Comparison of the sources of shape variation along this axis indicated whether *population source* or *salinity* more prominently explained the shape variation among mesocosm types (Table 3). For males, 48.5% of among-mesocosm type shape variation was explained by *population source*, compared to 9.4% by *salinity* and 1.6% by the *population source*×*salinity* interaction. For females, 39% of the variation was explained by *population source*, compared to 5.6% by *salinity* and 3.1% by the *population source*×*salinity interaction*. These results indicate that shape change associated with salinity change was rather small compared to natal population differences in shape. Further, the plasticity as visualized by the reaction norms (Figs. 2 and 3 and Figs. S1 and S2) was relatively limited for SS fish (i.e., the ancestral population for the refuge population at Mound Spring). These observations provide rather strong evidence that the Mound Spring deep-bodied shape was not a plastic response to lower salinity. Although the amount of variation explained by model effects was lower with the multivariate analysis – a result that is expected, since additional shape dimensions will reveal less information about inter-group shape differences [37] – population source remained largely more important than salinity as a source of shape variation.

### Genetic analyses

In contrast to the morphological dataset, data from 8 microsatellite markers showed no sign of divergence between Mound Spring and Salt Creek. Paired *F_ST_* (a measure of neutral divergence) between Mound Spring and Salt Creek was very low (0.019). Further, exact tests of sample differentiation found that Mound Spring was not significantly different from Salt Creek (*P* = 0.552). These results suggest that genetic drift within Mound Spring has been relatively modest, further supporting the adaptive nature of changes in body shape.

### Comparison of results to previous morphological analyses

The common garden revealed that the shape differences between Malpais Spring and Salt Creek pupfish raised in salinity mimicking their source habitat was similar to pupfish sampled from the habitats: Procrustes distance (*d*) was 0.185 and 0.221 for wild and mesocosm males, respectively; and 0.166 and 0.160 for both wild and mesocosm females, respectively. These similar Procrustes distances suggest that the mesocosm environments provided a reasonable match to conditions in the wild.

Procrustes distances for Salt Creek (SS) phenotypic plasticity were *d* = 0.117 and 0.109 for males and females, respectively. By comparison, the Procrustes distance between MM and SS pupfish were 0.233 and 0.248 for males and females, respectively.

## Discussion

Our results suggest that evolutionary divergence can occur within decades of population establishment. The observed divergence of the (deep-bodied) Mound Spring population was more than twice the divergence predicted from phenotypic plasticity (of the slender-bodied Salt Creek fish). Thus, the divergence of Mound Spring toward a deep body shape is not adequately explained by phenotypic plasticity. It is important to note that another non-native population introduced from Salt Creek to another saline creek (Lost River) in 1970 retained a streamlined body shape [11]. These data indicate that the Mound Spring population evolved a deeper body shape.

Based on three decades of isolation, divergence rates estimated as Haldanes [1] from the shape differences in wild populations were 0.174 sd/generation and 0.159 sd/generation for females and males, respectively. The divergence rates of the Mound Spring population exceed most rates recorded for vertebrates, especially for the observed interval of generations [1], [38]. Our data suggest that morphological divergence can occur in a small number of generations, which might explain why quantitative trait divergence exceeds molecular divergence for many other species of pupfishes [39]. Whether such large evolutionary divergence in body shape was adaptive requires examination of possible genetic drift. Genetic drift would leave a signature on allele frequencies of neutral loci such as microsatellites [40]–[42], which was not the case in our study, further supporting the hypothesis that the shape change was adaptive.

The evolutionary divergence of the Mound Spring population illustrates that contemporary evolutionary divergence of refuge populations is a biological phenomenon that should inform conservation plans. Our results are of particular interest as the White Sands pupfish is protected and the establishment of “refuge” populations is an important management tool [8], [43]. Such populations may actually diverge from the ancestral population during the time frame of a management plan (decades). Our results have broad relevance to conservation biology, showing that populations may evolve following assisted colonization, whether it be an intentional management response to climate change [44], or unintentional, as is the case with non-native species [2], [3].

We recognize that contemporary evolutionary divergence of introduced populations can be viewed as tool to enhance biodiversity; however, it is important to note that most introduced populations of desert fishes do not successfully establish [43]. Further, many factors might constrain evolutionary responses, not allowing the “refuge” population to adapt to local environments [2]. Contemporary evolution might also thwart conservation practices when an invasive species rapidly adapts [2]–[5]; thus, constraining restoration opportunities for protected species.

If refuge populations do not fulfill their role as genetic replicates, then cases like the White Sands pupfish might best be viewed as evolutionary experiments [3]. It is difficult to predict whether such divergence means the Mound Spring population would not establish if reintroduced to the Salt Creek environment. In terms of conservation management, such an expectation might be too costly a gamble.

## Supporting Information

Figure S1 Graphical representation of shape variation for male *C. tularosa*. Values are shape means projected on the first two principal components (PC) of among-group shape variation (representing 69.9% and 14.0% of among-group variation). Groups are the different source populations raised in either low or high salinity. Circles represent native crosses and squares represent hybrids. Solid symbols represent mesocosms that used Salt Creek females for the cross; open symbols represent mesocosms that used Malpais Spring females. Deformation grids are scaled 3×, and are presented to facilitate an understanding of shape differences. High Salinity (H) and low salinity (L) means are labeled.
(TIFF)

Figure S2 Graphical representation of variation (first two PCs) for female *C. tularosa*.
(TIFF)

Table S1 Summary of pupfish examined for morphometric data.
(PDF)

Appendix S1 Statistical and analytical details (plus references).
(PDF)
