# Supplementary figures and images for: Contemporary Evolutionary Divergence for a Protected Species following Assisted Colonization
Source: PLoS One. 2011 Aug 31;6(8):e22310. doi: 10.1371/journal.pone.0022310 (PMC3166134; doi:10.1371/journal.pone.0022310)

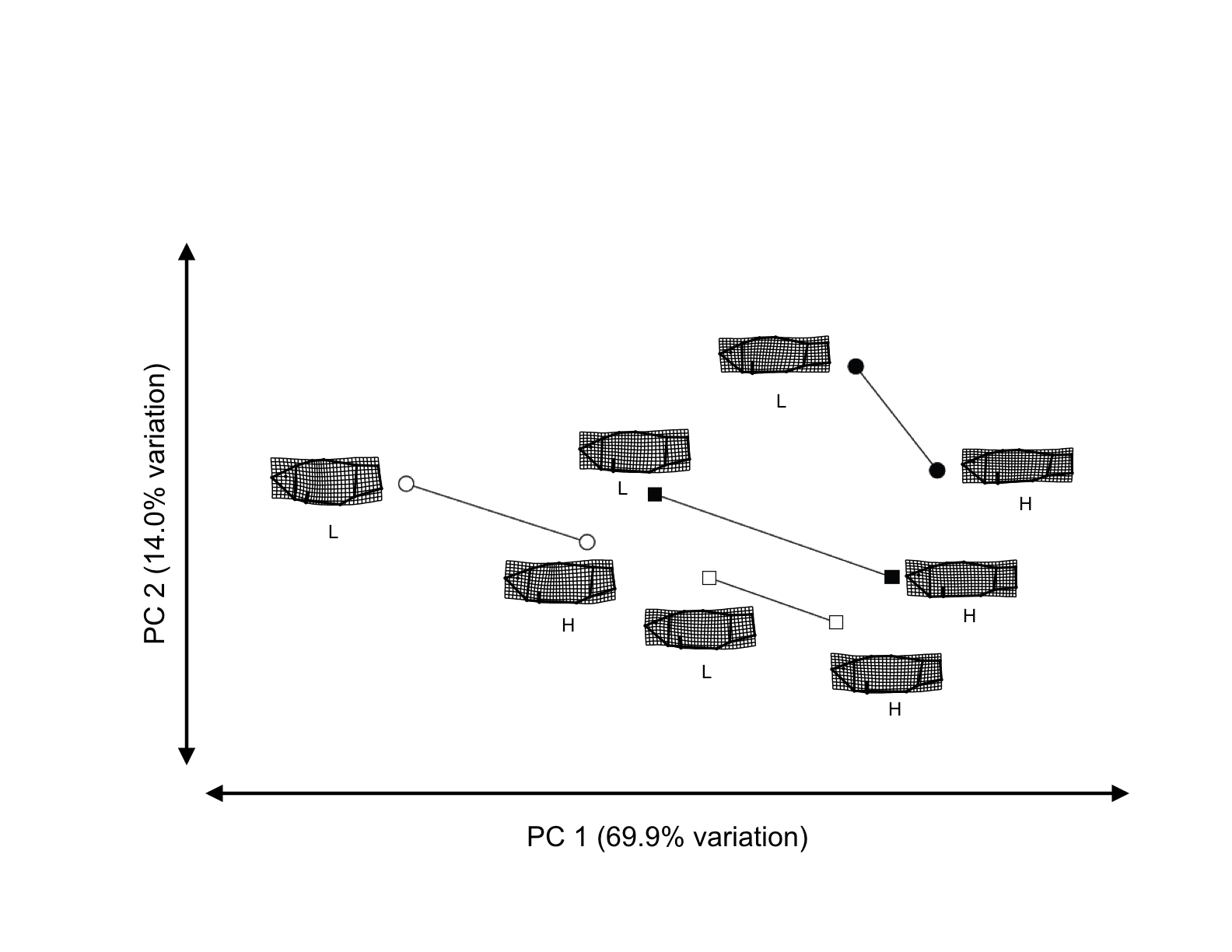

Supplement: Figure S1 — Graphical representation of shape variation for male C. tularosa. Values are shape means projected on the first two principal components (PC) of among-group shape variation (representing 69.9% and 14.0% of among-group variation). Groups are the different source populations raised in either low or high salinity. Circles represent native crosses and squares represent hybrids. Solid symbols represent mesocosms that used Salt Creek females for the cross; open symbols represent mesocosms that used Malpais Spring females. Deformation grids are scaled 3×, and are presented to facilitate an understanding of shape differences. High Salinity (H) and low salinity (L) means are labeled. (TIFF) [file pone.0022310.s001.tif]

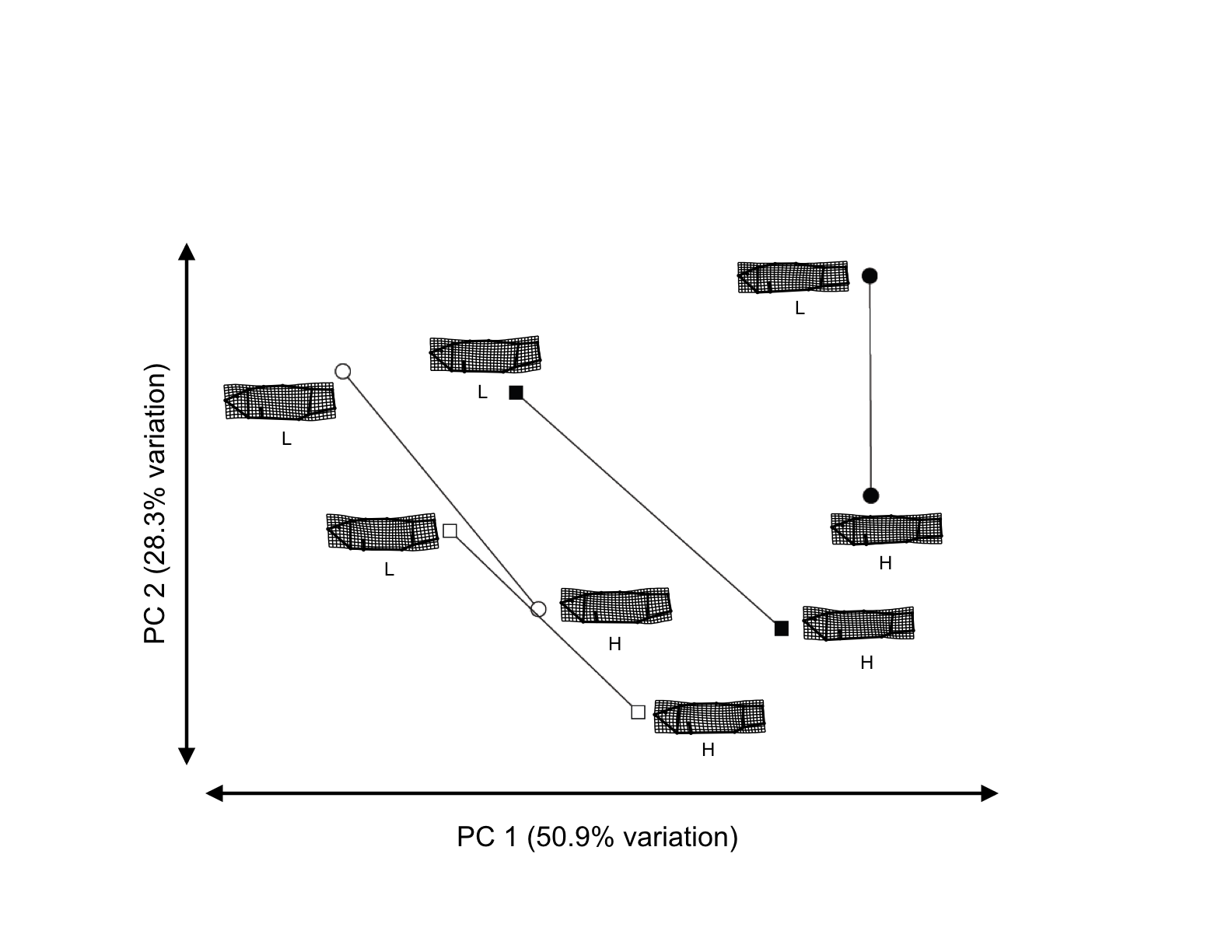

Supplement: Figure S2 — Graphical representation of variation (first two PCs) for female C. tularosa. (TIFF) [file pone.0022310.s002.tif]
