# Supplementary material for: Contemporary Evolutionary Divergence for a Protected Species following Assisted Colonization
Source: PLoS One. 2011 Aug 31;6(8):e22310. doi: 10.1371/journal.pone.0022310 (PMC3166134; doi:10.1371/journal.pone.0022310)
Supplement: Table S1 — Summary of pupfish examined for morphometric data. (PDF) [file pone.0022310.s003.pdf]

Table S1. Summary of pupfish examined for morphometric data. Crosses are denoted by the female-male combination of founders used to create mesocosm populations: M = Malpais Spring; S = Salt Creek. Experimental pool population size of adult pupfish is provided for each tank. The number of fish sampled (Female *n* plus Male *n*) was not always as large as the pool density (i.e., some fish were not preserved). Centroid size (CS) is the square root of the summed squared distances of the anatomical landmarks (Fig 1) to the configuration centroid [24].

Pool number	Cross	Salinity	Sex	*n*	"Density"	*min. CS*	*max. CS*
1	SS	L	F	9		23.9	37.69
1	SS	L	M	7	34	31.57	39.35
2	MM	L	F	22		25.04	32.22
2	MM	L	M	9	46	24.71	37.28
3	MM	H	F	4		30.64	39.08
3	MM	H	M	4	12	35.65	38.53
4	MS	L	F	4		27.66	39.02
4	MS	L	M	5	10	31.19	37.58
6	SS	H	F	10		26.52	34.71
6	SS	H	M	9	24	28.66	48.55
7	SM	L	F	4		27.83	40.45
7	SM	L	M	4	23	37.94	41.68
8	SM	H	F	5		26.89	35.49
8	SM	H	M	7	13	30.15	37.44
9	MM	L	F	3		26.82	43.58
9	MM	L	M	1	6	44.17	44.17
10	SS	L	F	14		24.83	32.5
10	SS	L	M	3	17	29.61	35.22
11	SS	H	F	20		22.76	37.93
11	SS	H	M	6	30	34.09	38.83
12	MM	H	F	14		24.69	38.1
12	MM	H	M	8	28	30.22	43.8
13	SS	H	F	9		25.06	35.14
13	SS	H	M	9	19	28.5	45.47
14	SS	L	F	7		27.42	32.34
14	SS	L	M	5	13	29.32	34.79
15	MS	H	F	3		26.14	39.65
15	MS	H	M	1	5	36.21	36.21
16	MM	H	F	14		25.01	30.82
16	MM	H	M	6	21	26.29	37.19
17	MS	L	F	15		24.31	31.59
17	MS	L	M	5	32	25.79	29.23
18	MM	L	F	1		38.03	38.03
18	MM	L	M	3	4	29.13	38.42
19	SM	H	F	16		24.58	30.91
19	SM	H	M	15	52	24.68	34.84
21	SM	L	F	3		31.79	38.48
21	SM	L	M	2	5	33.32	36.8
22	MM	H	F	15		21.33	41.09
22	MM	H	M	5	36	30.1	40.69
23	MM	L	M	2	2	43.24	45.64
24	SS	L	F	1		33.64	33.64
24	SS	L	M	2	3	35.64	36.17
25	MM	L	F	7		24.7	33.94
25	MM	L	M	3	16	28.03	31.05
26	SS	H	F	10		23.74	35.16
26	SS	H	M	6	23	25.1	37.57
27	MM	H	F	18		22.67	34.47
27	MM	H	M	7	32	30.13	32.5
28	SS	L	F	6		24.88	35.88
28	SS	L	M	6	12	31.22	38.79
29	MS	H	F	9		25.6	33.33
29	MS	H	M	11	43	25.55	32.73
30	MS	L	F	12		23.85	32.79
30	MS	L	M	8	23	29.4	33.4
31	SM	L	F	11		24.13	31.53
31	SM	L	M	9	26	25.84	34.12
32	SM	H	F	20		25.9	36.66
32	SM	H	M	18	40	26.45	35.91
33	SS	H	F	11		25.63	29.97
33	SS	H	M	8	56	25.94	35.53
34	MM	L	F	7		26.28	31.64
34	MM	L	M	3	12	31.17	46.11
35	MM	H	F	10		23.13	34.04
35	MM	H	M	5	22	29.07	42.26
36	SS	L	F	10		24.66	29.87
36	SS	L	M	15	33	23.62	31.23
