# Supplementary material for: Contemporary Evolutionary Divergence for a Protected Species following Assisted Colonization
Source: PLoS One. 2011 Aug 31;6(8):e22310. doi: 10.1371/journal.pone.0022310 (PMC3166134; doi:10.1371/journal.pone.0022310)
Supplement: Appendix S1 — Statistical and analytical details (plus references). (PDF) [file pone.0022310.s004.pdf]

Appendix S1: Statistical and Analytical Details

Collyer, Heilveil, and Stockwell

<u>Pool-adjusted shape values</u>

Univariate and multivariate analyses of variance (ANOVA and MANOVA, respectively) rely on linear models for estimation of fixed and random effects. Multivariate test statistics (e.g., Wilks' Λ) for MANOVA– which do not have proper distributions under null hypotheses – must be approximated by *F* statistics to assess significance [1]. Problems arise, however, in low-dimensional cases (e.g., when the number of response variables exceeds the number of responses, or when the number of model parameters is large compared to the number of responses) because there is no solution for conversion of multivariate test statistics to *F* statistics in some cases [1] and type-I error rates are exceedingly high for small subject-variable ratios [2]; nevertheless, effects can still be estimated by linear models, and appropriate statistical tests can also still be evaluated using a portion of model effects.

Our data suffered from low-dimensionality because the number of pools was smaller than the number of fixed effects. This problem was alleviated by adjusting individual fish shapes by their pool effects. The linear model for shape analyses in our study had the form $\mathbf{y}_{ijkl} = \boldsymbol{\mu} + size + \mathbf{p}_i + \mathbf{s}_j + \mathbf{ps}_{ij} + \mathbf{pool}_k \mid \mathbf{ps}_{ij} + \boldsymbol{\varepsilon}_{ijkl}$, meaning that any $1 \times p$ vector of p shape values ($\mathbf{y}$) for l^{th} individual fish in the ith population and j^{th} salinity treatment, assigned to the kth pool, is described by the overall mean ($\boldsymbol{\mu}$), plus corresponding population ($\mathbf{p}$), salinity ($\mathbf{s}$), and interaction ($\mathbf{ps}$) fixed effects, plus the pool random effect (nested within the population × salinity interaction), plus the covariate, size

(measured as log centroid size – see article). (Note that bold values refer to vectors.) The vector, $\boldsymbol{\varepsilon}_{ijkl}$, is a 1×*p* vector of residuals. The linear model can be written in matrix form as $\mathbf{Y} = \mathbf{X}\boldsymbol{\beta} + \boldsymbol{\varepsilon}$ to summarize that the *n* vectors of shape values in the *n*×*p* matrix, **Y**, are a function of covariates and dummy variables in the *n*×*k* design matrix, **X**, for the *k* parameters needed to describe the linear model above. The *k*×*p* matrix of regression coefficients, **β**, describes the effects of each parameter in the model, and the *n*×*p* matrix, **ε**, is a matrix of residuals. Parameter effects can be estimated as $\hat{\boldsymbol{\beta}} = \left(\mathbf{X}^{\mathrm{T}}\mathbf{X}\right)^{-1}\left(\mathbf{X}^{\mathrm{T}}\mathbf{Y}\right)$, where the superscripts, $^{\mathbf{T}}$, and $^{\mathbf{-1}}$, refer to matrix transpose and inverse, respectively [3]. Any estimated shape is thus solved as $\hat{\mathbf{y}} = \mathbf{x}\hat{\boldsymbol{\beta}}$, where **x** is a vector of appropriately coded parameters.

An important property of $\hat{\boldsymbol{\beta}}$ is that any estimated parameter effect is calculated with respect to other effects in the model. For example, population effects are estimated with respect to pool effects. Shape values can thus be estimated for only effects of interest without excluding extraneous sources of shape variation. Let matrix, $\hat{\boldsymbol{\beta}}'$, be a matrix of coefficients found in $\hat{\boldsymbol{\beta}}$, but with pool effects removed. This procedure is the same as mean-centering, if the intercept is removed [4]; therefore, the equation $\mathbf{Z} = \mathbf{X}\hat{\boldsymbol{\beta}}' + \boldsymbol{\varepsilon}$ creates adjusted shape values, **Z**, which have pool effects removed, but population and salinity effects are still estimated with respect to pool effects. A linear model of the form $\mathbf{z}_{ijkl} = \boldsymbol{\mu} + size + \mathbf{p}_i + \mathbf{s}_j + \mathbf{ps}_{ij} + \boldsymbol{\varepsilon}_{ijk}$ can be described such that individual fish, not pools are subjects, and $\boldsymbol{\varepsilon}_{ijk}$ does not inherently contain pool effects. Analyses of variance can be

performed on this model without the aforementioned statistical problems; however, one must be cautious that inflation of statistical power can result in incorrect inferences, [5].

In order to perform multivariate analysis of variance (MANOVA) without introducing problems of statistical power, we used a permutation procedure, which randomized individual shape values and recalculated model parameter estimates with each permutation. Sums of squares and cross products (SSCP) matrices were calculated for each effect [3] and the traces of these matrices – which are the total sums of squares for each effect – were used as test statistics. P-values were determined as percentiles of the observed test statistics in the empirical distributions of randomly generated statistics. This procedure has three key advantages. First, the test statistics are not plagued by improper degrees of freedom. Second, the test works equally well for univariate data, as the trace of a SSCP matrix is the sums of squares of the effect, itself. Third, R^2 values can be calculated for each effect, and can be compared within and between different analyses of univariate or multivariate shape data. We performed ANOVA and MANOVA for both male and female data sets using this method with 10,000 random permutations (the observed values constituting one permutation).

<u>Visualization of multivariate reaction norms</u>

Compared to univariate reaction norms in Figs 2 & 3, males and females had some differences in comparison of multivariate reaction norms, shown as vectors in the principal component plots of Figs S1 & S2. First, reaction norms were more aligned with the first PC for males, indicating that phenotypic plasticity in body shape was rather consistent with differences due to population source (Fig S1). Reaction norms for females indicated that

phenotypic plasticity was associated more so with the second PC, suggesting that body slendering in response to salinity was somewhat independent of evolved differences in body depth. For females more so than males, phenotypic plasticity in body shape for SS fish (principally along PC 2) was strikingly more independent of evolved differences in shape (principally along PC1). This visual result confirms why the *population source* × *salinity* interaction was significant in the MANOVA (Table 2) for females.

Although not obvious in the PC plots, differences between MM and SS body shapes were more pronounced in males. Procrustes distance, d, between SS high salinity fish and MM low salinity fish – the greatest inter-group difference – was 0.044 for males, compared to 0.027 for females. Females and males had similar ranges of phenotypic plasticities: Males: $0.013 \leq d \leq 0.024$ and Females: $0.012 \leq d \leq 0.021$. These results confirm the differences between males and females in terms of the ANOVA and MANOVA results. Males tend to have greater differences in shape based on population source. These results also suggest that the response to salinity changes is similar in scale for the two native strains; however, the differences in direction of shape change in the morphospace (Figs S1 and S2) indicate that the two populations might have different genetic covariances in the traits that make up body shape. Comparison of shape changes between pure Salt Creek fish (SS) and the other mesocosm types, between low and high salinity, suggests that SS fish deepen the dorsal aspect of the bodies in low salinity, whereas body deepening is more general for Malpais Spring (MM) fish and hybrids (either MS or SM).

Finally, maternal effects are more apparent in females, at least for hybrids from Malpais Spring female parents. MS hybrids were more similar to MM fish than SM fish. For both males and females, hybrid reaction norms were closer in the morphospace to the MM

reaction norm than the SS reaction norm, although this is more readily apparent for females. These results suggest that shape differences between MM and SS fish might be exacerbated by maternal effects. Based on the close proximity of MS and MM reaction norms, and the distinction of SM and SS reaction norms, females from less saline environments are prone to produce deep-bodied offspring but females from saline environments are not prone to produce slender-bodied offspring.

Multivariate AIC

The formula for multivariate AIC is given by Bedrick and Tsai [6] as

$\mathrm{AIC} = n\left[\ln\left(\frac{|\mathbf{E}|}{n^{p}}\right) + p\right] + 2\left[pk + 0.5p(p+1)\right]$, where n is the number of subjects, k is the number of model parameters, and p is the number of response variables. This equation has two parts: the first part express the log likelihood of a model, where $|\mathbf{E}|$ is the determinant of the sums of square and cross-products matrix of the model error; the second part is the parameter penalty. For univariate data the parameter penalty simplifies to $2(k + 1)$, which is often called $2K$ for convention [7].

Burnham and Anderson [7] recommended that a Δ AIC value of less than 2 suggests that neither of two competing models is sufficiently better than the other. This "rule of thumb", however, only applies to univariate response data. This value suggests that based on AIC scores, one would not expect two models with the same log-likelihoods to differ by more than one parameter. For example, if two models had the same log-likelihood but differed by one parameter (i.e., one model had $k + 1$ parameters compared to the k parameters of the other) for univariate response data, then Δ AIC =

$2\left[(k+1)+1\right]-2\left[k+1\right]=2$. Thus, a difference in AIC values of 2 more or less suggests that these models would not be expected to differ by more than one parameter if their log likelihoods were similar.

Using the multivariate development of this logic [6] for the *p* possible shape variables in a morphmetric study, two models with the same likelihood and which differ by one parameter would also differ by Δ AIC = 2*p*, because:

$$\mathrm{AIC}_1-\mathrm{AIC}_2=\left[n_1\left[\ln\left(\frac{|\mathbf{E}_1|}{n_1^p}\right)+p\right]+2[p(k+1)+0.5p(p+1)]\right]-\left[n_2\left[\ln\left(\frac{|\mathbf{E}_2|}{n_2^p}\right)+p\right]+2[p(k)+0.5p(p+1)]\right]$$

$$\approx 2\left[p(k+1)+0.5p(p+1)\right]-2\left[p(k)+0.5p(p+1)\right]$$

$$\approx 2\left[p(k+1)-pk\right]$$

$$\approx 2[pk+p-pk]$$

$$\approx 2p$$

Thus, it is difficult to either use the "rule of thumb" frequently used by many in model comparisons or to compare results of model comparisons between different numbers of principal components used to describe shape variables. The "rule of thumb" scales proportionally to the number of shape variables used; therefore, multiplying AIC values by 1/*p* nullifies this scaling, i.e., $\mathrm{AIC}^{*}=n\left[\frac{\ln\left(\frac{|\mathbf{E}|}{n^p}\right)}{p}+1\right]+2k+p+1.$

Applying the same logic (of same log likelihoods) yields $\mathrm{AIC}_1-\mathrm{AIC}_2\approx 2(k+1)-2k=2.$ We used this adjustment of AIC so that univariate and multivariate models would be more comparable.

Literature Cited for Appendix S1

1. Rencher, AC (2002) Methods of multivariate analysis. New York: Wiley.
2. Elliot, RS, Barcikowski, RS (1993) Multivariate test statistics and their approximations: some problems. Education Resources Information Center, report #ED359231.
3. Rencer, AC, Schaalje, GB (2008) Linear models in statistics. New York: Wiley.
4. Sokal, RR, Rohlf, FJ (1995) Biometry, 3rd ed. San Francisco: Freeman.
5. Hurlbert, SH (1984) Pseudoreplication and the design of ecological field experiments. Ecological Monographs 54:187-211.
6. Bedrick, EJ, Tsai, C (1994) Model selection for multivariate regression in small samples. Biometrics 50:226-231.
7. Burnham, KP, Anderson, DR (2002) Model selection and multimodel inference: a practical information-theoretic approach, 2nd Edition. New York: Springer-Verlag.
